# Layered PLLA with monopolar RF for facial rejuvenation: a case report

**DOI:** 10.1080/23320885.2026.2748235

**Published:** 2026-10-14

**Authors:** Xi Yang, Ling Tang, Juntian Liu, Ying Liu

**Affiliations:** Shanghai Zhimei Medical Beauty Outpatient Department Co., Ltd, Shanghai, China

**Keywords:** Poly-L-lactic acid, monopolar radiofrequency, facial rejuvenation, combination therapy, layered injection

## Abstract

With the continuous expansion of facial rejuvenation techniques, the combination of energy-based devices and biostimulatory fillers has become a focal point in clinical research. Monopolar radiofrequency (MRF), such as Thermage, can achieve immediate tissue tightening, while poly-L-lactic acid (PLLA), a collagen stimulator, enables volumetric restoration and lifting through progressive collagen regeneration. This article describes a sequential treatment protocol combining MRF followed by layered PLLA injection in three patients with facial laxity and volume loss. Clinical photographs and Wrinkle Severity Rating Scale (WSRS) assessments were collected during follow-up. A split-face approach was applied in Case 1, with combination therapy (MRF + PLLA) administered to the more affected side and MRF alone to the contralateral side. Improvements in facial contour appearance, nasolabial fold severity, and facial symmetry were observed during follow-up. No treatment-related adverse events were reported during the observation period. These cases provide preliminary short-term clinical observations regarding this sequential treatment strategy; however, the contribution of each modality cannot be distinguished and further controlled studies are required.

## Introduction

In the field of facial rejuvenation, the combined use of energy-based devices and injectable products is garnering increasing clinical attention [1,2]. Monopolar radiofrequency (MRF) delivers high-frequency energy that induces dermal heating, leading to collagen contraction and subsequent neocollagenesis, thereby producing an immediate tissue-tightening effect [3]. Meanwhile, PLLA progressively promotes the synthesis of type I and type III collagen, achieving volume restoration and tissue remodeling [4]. Both MRF and PLLA exert skin-tightening effects; however, PLLA provides a longer-lasting response and additionally promotes natural volumetric restoration and skin texture improvement, supporting a complementary therapeutic profile [5,6]. However, systematic studies for combined MRF and PLLA are still limited. We therefore tested a fixed‑sequence protocol in a small series, consisting of ‘MRF first, followed by layered PLLA injection’, aiming to document preliminary clinical observations regarding this treatment sequence and to describe its short-term procedural experience in routine practice. In three typical cases (one with a split‑face design), this approach appeared to be feasible, and our preliminary findings might offer some useful reference for clinical practice, though further study is warranted.

## Case report

This study included three female patients, all presenting with facial skin laxity accompanied by volume loss. They had not undergone any other aesthetic procedures within the preceding 12 months, had no relevant medical history or underlying systemic diseases, and all provided signed informed consent. The total treatment protocol consisted of one MRF session and two PLLA injection sessions per patient. All three patients underwent full-face MRF followed one month later by the first PLLA injection, and then, after an additional one‑month interval, a second PLLA injection was administered (Case 1 received combination treatment on the right side, with the left side receiving MRF only; Cases 2 and 3 received full-face combination treatment). Two-dimensional photography was performed for all patients before treatment, one month after the first PLLA injection, and three months after the second PLLA injection. For these photographs, the distance, facial expression, and head position were standardized across all time points. Although efforts were made to standardize photography, lighting conditions were not completely identical across visits. This limitation should be considered when interpreting visual comparisons, and future studies should employ fully standardized photographic systems. A blinded physician independently evaluated and documented the treatment outcomes of three patients before and after treatment, using the Wrinkle Severity Rating Scale (WSRS). Treatment-related adverse events, including pain, erythema, edema, bruising, nodules, and delayed inflammatory reactions, were monitored through immediate post-treatment examination and active questioning and local examination at routine follow-up visits. Patients were also asked about transient reactions that had resolved before follow-up and could report any symptoms or concerns between visits. All reported or observed events were documented in the medical records.

### Case 1 (Patient A, 25 years old)

The patient presented with facial skin laxity accompanied by asymmetry of the nasolabial folds, with the right side demonstrating greater depth and volume deficiency compared to the left. In light of the baseline asymmetry, we felt that a split‑face approach with differential treatment might be useful, in the hope that it could help improve overall facial balance in this case. After initial full-face MRF (5th generation Thermage CPT, 900 pulses, treatment depth 4.5–5.0 mm), PLLA was first injected on the right side of the face one month later, for a total of two injections at one-month intervals. The PLLA product used was Löviselle (340 mg/vial, Changchun Sinobiomaterials Co., Ltd.), synthesized using PLLA-LaSynPro^™^ technology. Reconstitution method: Each vial of PLLA lyophilized powder was reconstituted with sterile saline to a volume of 3 mL. A 25 *G* × 50 mm cannula was used for layered injection. In Case 1, PLLA was injected only on the right side of the face, with a volume of 6 mL (2 vials) per session, for a total of 12 mL (4 vials) over the two sessions. Injection points and layers were as follows (Figure 1):

**Figure 1.**
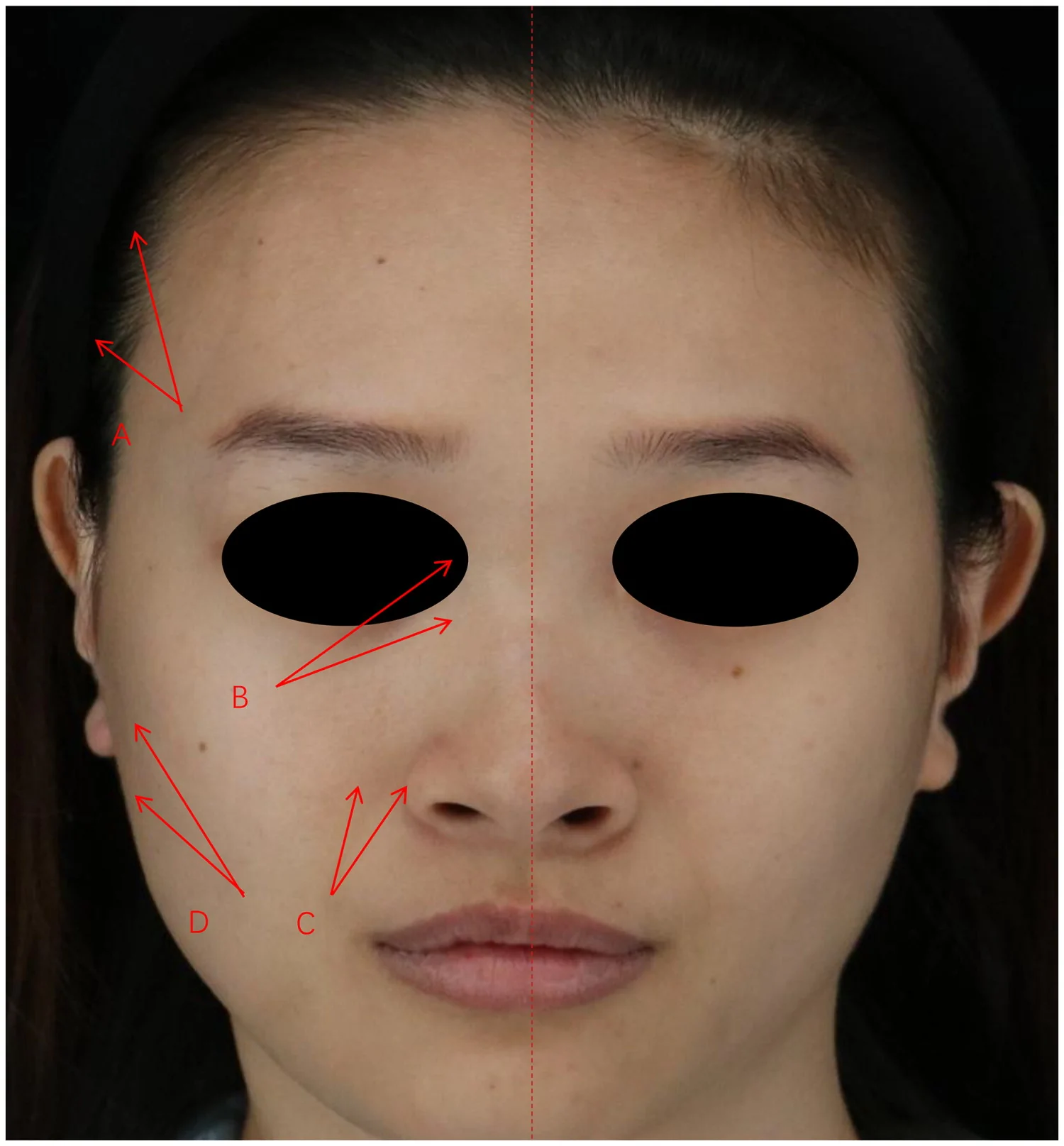
Injection sites of Poly-L-lactic acid injection for facial rejuvenation.

- Point A: Deep layer of temporal fascia, injection volume 2 mL
- Point B: SOOF area and superficial fat pad, injection volume 2 mL
- Point C: Deep and superficial fat pads, injection volume 1 mL
- Point D: Subcutaneous layer, injection volume 1 mL

Injection volume of each single tunnel was 0.2–0.3 mL, using a fanning retraction injection technique to ensure uniform product distribution.

During follow-up, both sides demonstrated reductions in WSRS scores and improvement in overall facial appearance. Although the right side appeared to improve earlier in some features, interpretation is limited because baseline asymmetry was present and the combination treatment was intentionally applied to the more affected side (Figure 2). On the right side, the WSRS score decreased from 3 at baseline to 2 at one month after the first treatment, and then to 1 at three months after the second treatment. On the left side, the score was 2 at baseline, remained 2 at one month after the first treatment, and decreased to 1 at three months after the second treatment, showing a reduction in WSRS scores on both sides during follow-up (Figure 3). Following this treatment strategy, the asymmetry of nasolabial folds between the left and right sides appeared to be reduced in this case. No adverse reactions were observed on either side.

**Figure 2.**
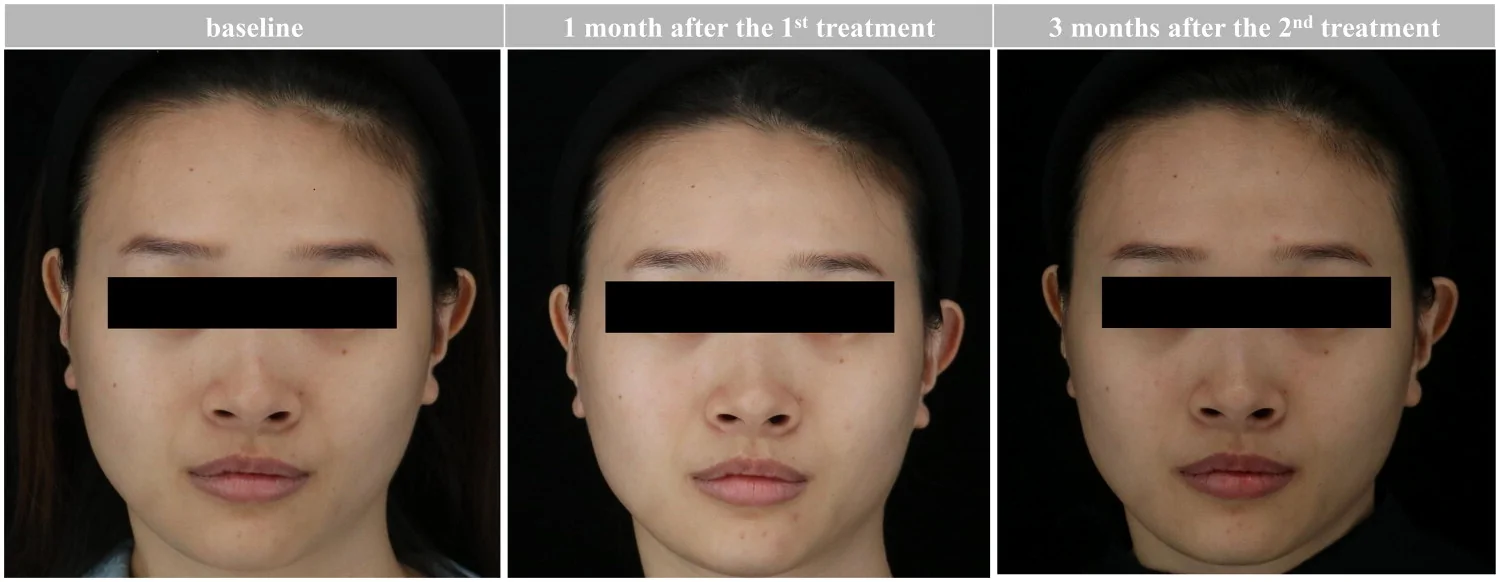
Clinical photographs of Case 1 at baseline, 1 month after the first PLLA injection, and 3 months after the second PLLA injection.

**Figure 3.**
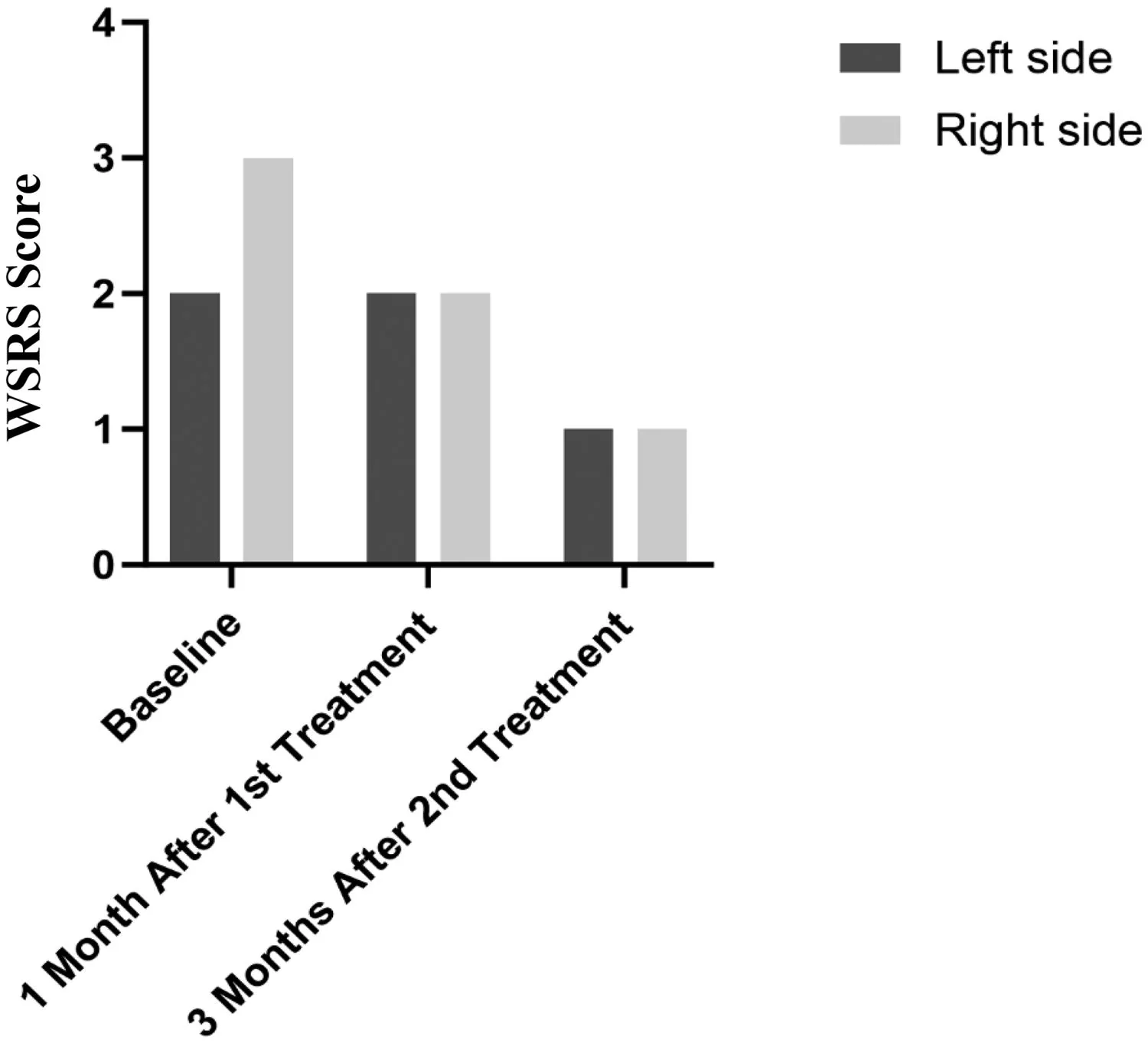
Changes in Wrinkle Severity Rating Scale (WSRS) scores for the left and right nasolabial folds in Case 1 at baseline, 1 month after the first treatment, and 3 months after the second treatment.

### Cases 2 and 3 (Patients B, 24 years old and C, 26 years old)

Both patients underwent full-face MRF followed by full-face layered PLLA injections. PLLA was injected bilaterally, with the same injection points and the same manipulation technique at each point on both sides (Figure 1). For each of these two patients, a volume of 6 mL (2 vials) was injected per side per session, corresponding to a full‑face volume of 12 mL (4 vials) per session. Over the two sessions, the cumulative total dose per patient was 24 mL (8 vials). In Case 2, one month after the first injection, we noted mild volumetric restoration in the tear trough and temporal area, together with some improvement in jawline contour. At the three-month follow-up after the second injection, the nasolabial folds appeared to be less deep, and lifting effects in the subzygomatic region and marionette lines were also noted, with an overall improvement in facial smoothness that was considered appreciable in this patient (Figure 4a). Patient C had milder baseline skin laxity, primarily presenting with mild facial looseness and deepening of the nasolabial folds. At the three-month follow-up after the second injection, the cheek area appeared to show improved fullness, with a certain degree of reduction in nasolabial folds and what seemed to be enhanced facial smoothness and tightness (Figure 4b). For Patient B, the bilateral WSRS scores were 2 at baseline, 1 at one month after the first treatment, and 1 at three months after the second treatment. For Patient C, the bilateral WSRS scores were 2 at baseline, 2 at one month after the first treatment, and 1 at three months after the second treatment, providing a more complete description of the WSRS changes during follow-up. No adverse events, such as nodules, persistent erythema, or infection, were observed during the treatment course for any of the three patients.

**Figure 4.**
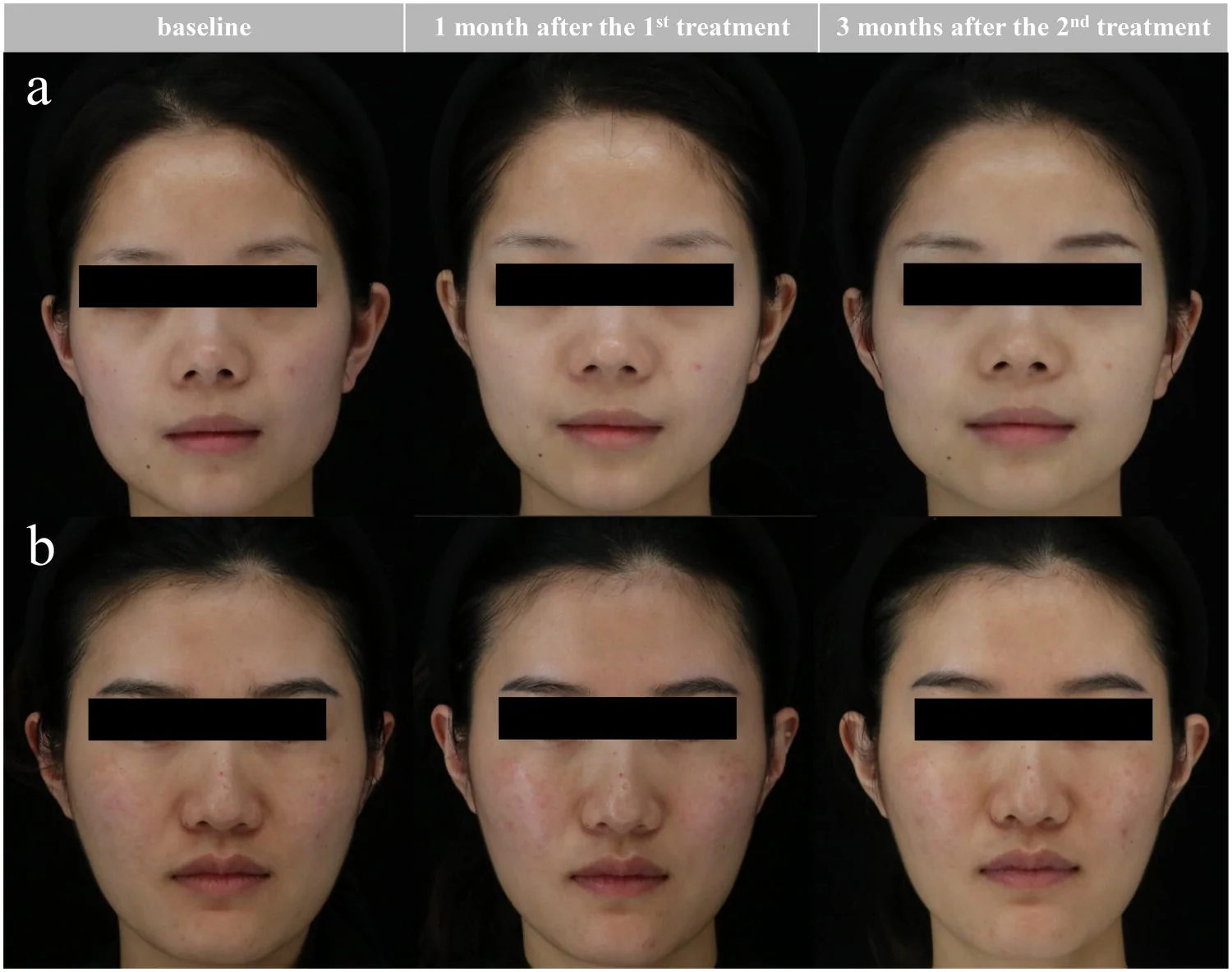
Clinical photographs of Case 2(a) and 3(b) at baseline, 1 month after the first PLLA injection, and 3 months after the second PLLA injection.

## Discussion

Multimodal facial rejuvenation strategies have attracted increasing attention because facial aging typically involves multiple structural components, including skin laxity, volume loss, and contour changes. Previous reports combining PDO threads with botulinum toxin A have demonstrated improvements in patient satisfaction, wrinkle appearance, and skin texture, suggesting that treatments targeting different aging mechanisms may provide complementary aesthetic benefits. Nevertheless, outcomes and durability vary substantially among modalities and patient populations, and findings from small uncontrolled studies should be interpreted cautiously [7–8]. The present report contributes only preliminary observations regarding a sequential MRF-PLLA approach and does not establish superiority or synergy between treatments.

The safety and sequencing considerations of combining energy‑based devices with injectable polymer materials have been explored in cadaveric models, underscoring the importance of understanding how such materials respond to thermal energy-a consideration that may also be relevant when combining MRF with PLLA [9]. In our protocol, the sequencing of MRF one month prior to the first PLLA injection was intended to allow sufficient time for the initial tissue response to MRF to subside before PLLA administration, thereby potentially minimizing any thermal interaction between the two modalities.

The cumulative PLLA volume used in this series reflected the full-face multilayer treatment strategy rather than an intention to maximize product dose. PLLA was distributed across multiple anatomical compartments, including the deep temporal fascia, SOOF, deep and superficial facial fat compartments, and subcutaneous tissues. Consequently, the reported volume represented the cumulative amount required to provide broad structural support across the face rather than concentrated augmentation of a single treatment area. The dosing regimen described in this report reflects the authors’ clinical approach in these specific cases and should not be interpreted as a standardized treatment recommendation. No nodules, persistent erythema, infection, or other treatment-related adverse events were reported during the follow-up period. However, the limited sample size and short duration of follow-up do not permit conclusions regarding the safety profile of this treatment strategy.

An important limitation of the present case series is that the respective contributions of MRF and PLLA cannot be separated. In Cases 2 and 3, both modalities were administered sequentially and no MRF-only comparator was available. Furthermore, delayed remodeling effects following MRF may have continued throughout the observation period. Consequently, the observed improvements cannot be attributed specifically to PLLA, MRF, or their interaction, and should instead be interpreted as outcomes observed after a predefined sequential treatment protocol. The split-face design in Case 1 was further limited by pre-existing nasolabial fold asymmetry, as the combination treatment was intentionally applied to the more affected side, restricting direct side-to-side comparison. In addition, the small number of cases and the absence of a control group preclude any definitive conclusions regarding efficacy, superiority, or synergistic effects of the combined approach. Future controlled studies with larger cohorts, longer follow-up, standardized patient-reported outcomes, and objective assessment tools are needed to further evaluate this treatment sequence and determine its potential role within multimodal facial rejuvenation strategies.

## Conclusion

This three-patient case series describes preliminary short-term observations following sequential treatment with monopolar radiofrequency and layered PLLA injection. Improvements in facial contour appearance, nasolabial fold severity, and facial symmetry were observed during follow-up, and no treatment-related adverse events were reported during the observation period. Larger controlled studies are needed to further evaluate this treatment approach.

## Supplementary Material

Attachments.zip

## Data Availability

The data supporting the findings of this study are available from the corresponding author upon reasonable request.
